# Effect of perindopril or leucine on physical performance in older people with sarcopenia: the LACE randomized controlled trial

**DOI:** 10.1002/jcsm.12934

**Published:** 2022-02-16

**Authors:** The LACE study group, Marcus Achison, Simon Adamson, Asangaedem Akpan, Terry Aspray, Alison Avenell, Margaret M. Band, Tufail Bashir, Louise A. Burton, Vera Cvoro, Peter T. Donnan, Gordon W. Duncan, Jacob George, Adam L. Gordon, Celia L. Gregson, Adrian Hapca, Emily Henderson, Cheryl Hume, Thomas A. Jackson, Paul Kemp, Simon Kerr, Alixe Kilgour, Veronica Lyell, Tahir Masud, Andrew McKenzie, Emma McKenzie, Harnish Patel, Kristina Pilvinyte, Helen C. Roberts, Christos Rossios, Avan A. Sayer, Karen T. Smith, Roy L. Soiza, Claire J. Steves, Allan D. Struthers, Deepa Sumukadas, Divya Tiwari, Julie Whitney, Miles D. Witham

**Affiliations:** ^1^ Tayside Clinical Trials Unit (TCTU) Tayside Medical Science Centre (TASC), University of Dundee, Ninewells Hospital & Medical School Dundee UK; ^2^ Clinical Research Network Northwest Coast University of Liverpool, Liverpool University Hospitals NHS FT Trust Liverpool UK; ^3^ AGE Research Group NIHR Newcastle Biomedical Research Centre, Translational Clinical Research Institute, Newcastle University and Newcastle‐upon‐Tyne NHS Trust Newcastle upon Tyne UK; ^4^ Health Services Research Unit University of Aberdeen Aberdeen UK; ^5^ Cardiovascular and Respiratory Interface Section National Heart and Lung Institute, Imperial College London, South Kensington Campus London UK; ^6^ Medicine for the Elderly NHS Tayside, Dundee, UK and Ageing and Health, University of Dundee Dundee UK; ^7^ Victoria Hospital Kirkcaldy UK; ^8^ Centre for Clinical Brain Sciences University of Edinburgh Edinburgh UK; ^9^ Division of Population Health and Genomics, School of Medicine University of Dundee Dundee UK; ^10^ Medicine for the Elderly NHS Lothian, Edinburgh, UK and Centre for Clinical Brain Sciences, University of Edinburgh Edinburgh UK; ^11^ Department of Clinical Pharmacology, Division of Molecular & Clinical Medicine University of Dundee Medical School, Ninewells Hospital Dundee UK; ^12^ Unit of Injury, Inflammation and Recovery, School of Medicine University of Nottingham Nottingham UK; ^13^ NIHR Nottingham Biomedical Research Centre Nottingham UK; ^14^ Department of Medicine for the Elderly University Hospitals of Derby and Burton NHS Foundation Trust Derby UK; ^15^ Musculoskeletal Research Unit Bristol Medical School, University of Bristol Bristol UK; ^16^ Older Person's Unit Royal United Hospital NHS Foundation Trust Bath Bath UK; ^17^ Population Health Sciences, Bristol Medical School University of Bristol Bristol UK; ^18^ Royal United Hospital Bath NHS Foundation Trust Bath UK; ^19^ Institute of Inflammation and Ageing, University of Birmingham Birmingham UK; ^20^ Department of Older People's Medicine Newcastle upon Tyne Hospitals NHS Foundation Trust Newcastle upon Tyne UK; ^21^ Medicine for the Elderly NHS Lothian Edinburgh UK; ^22^ Clinical Gerontology Research Unit Nottingham University Hospitals NHS Trust, City Hospital Campus Nottingham UK; ^23^ NIHR Biomedical Research Centre University of Southampton and University Hospital Southampton NHSFT Southampton UK; ^24^ Academic Geriatric Medicine University of Southampton, Mailpoint 807 Southampton General Hospital Southampton UK; ^25^ Ageing & Clinical Experimental Research (ACER) Group University of Aberdeen Aberdeen UK; ^26^ Department of Twin Research and Genetic Epidemiology, King's College London & Department of Clinical Gerontology King's College Hospital London UK; ^27^ Division of Molecular and Clinical Medicine University of Dundee, Ninewells Hospital Dundee UK; ^28^ Department of Medicine for the Elderly NHS Tayside Dundee UK; ^29^ Bournemouth University and Royal Bournemouth Hospital Bournemouth UK; ^30^ School of Population Health & Environmental Sciences King's College London and King's College Hospital London UK

**Keywords:** Angiotensin converting enzyme inhibitor, Leucine, Sarcopenia, Randomized controlled trial

## Abstract

**Background:**

This trial aimed to determine the efficacy of leucine and/or perindopril in improving physical function in older people with sarcopenia.

**Methods:**

Placebo‐controlled, parallel group, double‐blind, randomized two‐by‐two factorial trial. We recruited adults aged ≥ 70 years with sarcopenia, defined as low gait speed (<0.8 m/s on 4 m walk) and/or low handgrip strength (women < 20 kg, men < 30 kg) plus low muscle mass (using sex and body mass index category‐specific thresholds derived from normative UK BioBank data) from 14 UK centres. Eligible participants were randomized to perindopril 4 mg or placebo, and to oral leucine powder 2.5 g or placebo thrice daily. The primary outcome was the between‐group difference in the short physical performance battery (SPPB) score over 12‐month follow‐up by repeated‐measures mixed models. Results were combined with existing systematic reviews using random‐effects meta‐analysis to derive summary estimates of treatment efficacy.

**Results:**

We screened 320 people and randomized 145 participants compared with an original target of 440 participants. For perindopril [*n* = 73, mean age 79 (SD 6), female sex 39 (53%), mean SPPB 7.1 (SD 2.3)] versus no perindopril [*n* = 72, mean age 79 (SD 6), female sex 39 (54%), mean SPPB 6.9 (SD 2.4)], median adherence to perindopril was lower (76% vs. 96%; *P* < 0.001). Perindopril did not improve the primary outcome [adjusted treatment effect −0.1 points (95%CI −1.2 to 1.0), *P* = 0.89]. No significant treatment benefit was seen for any secondary outcome including muscle mass [adjusted treatment effect −0.4 kg (95%CI −1.1 to 0.3), *P* = 0.27]. More adverse events occurred in the perindopril group (218 vs. 165), but falls rates were similar. For leucine [*n* = 72, mean age 78 (SD 6), female sex 38 (53%), mean SPPB 7.0 (SD 2.1)] versus no leucine [*n* = 72, mean age 79 (SD 6), female sex 40 (55%), mean SPPB 7.0 (SD 2.5)], median adherence was the same in both groups (76% vs. 76%; *P* = 0.99). Leucine did not improve the primary outcome [adjusted treatment effect 0.1 point (95%CI −1.0 to 1.1), *P* = 0.90]. No significant treatment benefit was seen for any secondary outcome including muscle mass [adjusted treatment effect −0.3 kg (95%CI −1.0 to 0.4), *P* = 0.47]. Meta‐analysis of angiotensin converting enzyme inhibitor/angiotensin receptor blocker trials showed no clinically important treatment effect for the SPPB [between‐group difference −0.1 points (95%CI −0.4 to 0.2)].

**Conclusions:**

Neither perindopril nor leucine improved physical performance or muscle mass in this trial; meta‐analysis did not find evidence of efficacy of either ACE inhibitors or leucine as treatments to improve physical performance.

## Introduction

Sarcopenia is associated with a broad range of important adverse consequences for older people including falls, fractures, increased risk of hospitalization, longer length of hospital stay, an increased risk of needing care including institutional care, and earlier death.^1^, ^4^ Sarcopenia is common, affecting 5–10% of the population aged over 65, with rates exceeding 30% in settings such as care homes or in hospital.^5^, ^6^ The cost of sarcopenia to the UK health economy has been estimated at approximately £2 billion each year.^7^

Resistance training is the intervention with the most evidence to prevent or reverse sarcopenia^6^, ^8^, ^9^; however, not all older people with sarcopenia are either willing or able to undertake resistance training. Access to resistance training is insufficient to meet demand given the burden of sarcopenia on the older population.^10^ New ways to prevent and treat sarcopenia are therefore required to alleviate the significant health burden caused by this condition. It is now clear that sarcopenia is a complex and multifactorial disorder involving a range of fundamental biological processes including cellular senescence, chronic inflammation, neurohormonal dysregulation, vascular dysfunction, neuromuscular junction deficits, oxidative stress and mitochondrial dysfunction, and deficits in muscle protein uptake and utilization.^11^ The relative contributions of these pathologies remain to be fully delineated.

Preliminary data suggest that derangements of the renin angiotensin aldosterone system may be important in the pathology of sarcopenia. Observational data show that older people taking angiotensin converting enzyme inhibitors (ACEi) have better preserved walk speed over a 3‐year period than those not taking ACEi.^12^ Some randomized controlled trials have suggested a beneficial effect of ACEi compared with placebo on physical performance in older people although other studies have failed to find such beneficial effects, and a recent systematic review did not find an overall benefit of either ACEi or angiotensin receptor blockers (ARBs) in this age group.^13^ The conclusions of this review were limited by the lack of studies specifically enrolling older people with sarcopenia. Similarly, some studies suggest that leucine supplementation may have beneficial effects on muscle protein synthesis, uptake of amino acids, and consequent improvement in muscle strength.^14^, ^16^ However, very few studies have set out to examine the effect of leucine in older people with sarcopenia as opposed to younger people or healthy older people.^17^

Therefore, there is a clear need for randomized controlled trials to test the efficacy of ACEi and of leucine in older people with sarcopenia. In this trial, we aimed to test the effects of 1 year of treatment with the ACEi perindopril versus placebo, and of leucine supplementations versus placebo, in a population of older people with sarcopenia who were not undergoing resistance training.

## Methods

LACE (Leucine or Angiotensin Converting Enzyme inhibitors for sarcopenia) was a parallel group, double‐blind, placebo‐controlled, randomized 2 × 2 factorial trial. Participants were eligible for inclusion if they were aged 70 years and over and had sarcopenia according to the European Working Group on Sarcopenia in Older People (EWGSOP) 2010 definition of sarcopenia, which requires the presence of low muscle strength or low physical performance, plus low muscle mass.^18^ Definitions for low muscle strength and low physical performance followed the EWGSOP 2010 sarcopenia guidelines, in which the presence of either gait speed < 0.8 m/s on 4 m walk (low physical performance) and/or low handgrip strength (<20 kg for women, <30 kg for men) was required for case identification. Gait speed is strongly associated with survival, and a value of 0.8 m/s is associated with the median survival time in a pooled analysis of observational studies.^19^ The handgrip cut‐offs used were derived from a population‐based observational study and were found to be optimal to discriminate participants with mobility limitation.^20^

For muscle mass, we used height‐adjusted total skeletal muscle mass measured by Bioimpedance Assay (BIA) using the Akern BIA 101 device and the Sergi equation.^21^ Cut‐offs varied with body mass index and sex to ensure that participants with sarcopenic obesity could be recruited. Table S1 shows the body mass index and sex‐specific muscle mass cut‐offs used at screening. These cut‐offs were derived from normative bioimpedance data from healthy middle aged adults in the UK BioBank study^22^ Total body fat‐free mass index cut‐offs for the fifth percentile were used and converted to equivalent appendicular skeletal muscle mass values using values from Kyle *et al*.^23^

Trial exclusion criteria were selected to (a) avoid contraindications to ACE inhibitors, (b) avoid contraindications to key outcomes or inability to consent, and (c) exclude participants with skeletal myopathy clearly due to an alternative cause rather than sarcopenia. A full list of exclusion criteria is given in Table S2. The trial protocol has been published previously.^24^ The trial was approved by the East of Scotland NHS research ethics committee (approval 14/ES/1099) and the UK Medicines and Healthcare Regulatory Authority (EudraCT number 2014‐003455‐61; Clinical Trial Authorisation number 36888/0001/001‐0001); the trial was performed in accordance with the ethical standards laid down in the 1964 Declaration of Helsinki and its later amendments.

### Study interventions

The trial intervention consisted of either perindopril erbumine (KRKA Polska, Warsaw, Poland and TEVA pharmaceuticals, Peta Tikva, Israel), overencapsulated with a gelatine capsule packed with microcrystalline cellulose, or matching placebo capsules packed only with microcrystalline cellulose. Active and placebo tablets were manufactured and bottled by Tayside Pharmaceuticals, Dundee, UK, who undertook quality testing, qualified person release, and distributed bottles to participating sites. Study medications were held at site pharmacies under temperature‐controlled conditions prior to dispensing to participants. For the first 2 weeks of participation, participants were given capsules containing perindopril 2 mg or placebo and instructed to take one capsule per day. If uptitration occurred at 2 weeks, a fresh supply of capsules was dispensed, containing 4 mg of perindopril or placebo; participants were again instructed to take one capsule per day. A flowchart depicting the titration schedule is given in Figure S1.

Bulk leucine powder was obtained from Amino GmbH (Freilstedt, Germany). Study pots (one pot per participant per month) were prepared by Tayside Pharmaceuticals, Dundee, UK, who undertook quality testing, qualified person release, and distributed pots to participating sites. Leucine/placebo pots were held at site pharmacies under temperature‐controlled conditions prior to dispensing to participants. Pots contained either 400 g of leucine powder, or 400 g of lactose powder, selected for its similarity of appearance. Participants were supplied with 1.5‐mL scoops and were asked to ingest three scoops of powder three times a day, with meals, equivalent to 2.5 g of leucine three times a day, a daily total of 7.5 g. Participants were encouraged to mix the powder with drinks or yoghurts or spread the powder on food; serving suggestions were shared with participants at the start of their participation.

Randomization and treatment allocation were performed remotely using an interactive web‐based randomization, drug assignment, and inventory management system (TRuST) run by the Health Informatics Centre, University of Dundee. The system was run independently from the research team to preserve allocation concealment. Randomization was performed in a 1:1 ratio for both perindopril/placebo and leucine/placebo, stratified by site, and employed a minimization algorithm with a small random element using the following minimization factors: age, sex, short physical performance battery (SPPB), Charlson score,^25^ and grip strength. Participants were allocated study medication bottles with either perindopril capsules or matching placebo, and tubs containing 400 g of leucine or matching placebo. Bottles and tubs were allocated based on bottle ID numbers generated by the TRuST randomization system and were not labelled with any indication of whether they contained the active or placebo substance.

Adherence to perindopril or placebo was ascertained by tablet counting, with adherence calculated as number of tablets taken/number of tablets scheduled to be taken between baseline and study completion or dropout. Leucine/placebo adherence was checked by weighing container tubs at each safety visit, with adherence calculated as weight of powder used/weight expected to be used between baseline and study completion or dropout.

### Outcomes

Outcomes visits were conducted at baseline, 6 and 12 months, with additional visits for research bloods at 3 months and adverse event recording at 9 months. An additional visit for safety bloods and uptitration of perindopril/placebo took place at 2 weeks, with further safety bloods at 5 weeks. Outcomes were collected by research nurses at each site who were masked to treatment allocation. The primary outcome was the between‐group difference in the SPPB score across the follow‐up period. The SPPB was measured at baseline, 6 and 12 months. The SPPB tests lower limb strength and balance^26^ and consists of three tests—a balance test (side by side balance, semi‐tandem balance, and tandem balance), a timed sit to stand from a chair five times and walk speed over a 4‐m course. The test is scored from 0 (worst; includes those who cannot perform any component) to 12 (best score). The SPPB is a robust predictor of a range of adverse outcomes in older people including death, dependency, and future disability.^26^, ^27^

### Secondary outcomes

Table S3 lists the secondary outcomes measured as part of the LACE trial. All secondary outcomes were assessed as the between‐group difference in each measure across the follow‐up period. Data were collected on falls using monthly prospective falls diaries and on diet at baseline using the Scottish Collaborative Group Food Frequency Questionnaire^28^ to examine total protein intake.

### Sample size calculation

The minimum clinically important difference (MCID) for the SPPB has been estimated at between 0.5 and 1 point.^27^, ^29^ We took a deliberately conservative approach to sample size calculation and used an MCID of 0.5 points difference between the treatment arms at 12 months. Assuming a standard deviation of 2.7 as seen in similar previous studies with a power of 90% at alpha 0.05, and assuming a correlation between time points of 0.7 as seen from our previous work, we would require 88 participants in each of the four groups (352 participants in total) to detect this MCID at 12 months. Assuming 20% dropout at 12 months (based on previous similar studies^30^, ^31^), we therefore aimed to recruit 440 patients. This sample size would also have 90% power to detect a 5% difference in muscle mass, assuming a baseline value of 19 kg (SD 2.8). Sample size calculations were performed using NQuery Adviser software v7.0 (StatSols, Cork, Ireland).

### Statistical analysis

Analyses were performed using Statistical Analysis System (SAS) v9.4 software (SAS Institute Inc, Cary, NC, USA) using modified intention‐to‐treat methods. Unmasking of randomization groups was performed only after completion of the statistical analysis. A two‐sided *P* value of <0.05 was taken as significant for all analyses, with no adjustment for multiple testing. A mixed effect linear model was used to analyse the primary analysis response variable (SPPB score data from 6 and 12 months) with variables treatment (treatment vs. no treatment), SPPB at baseline, age, sex, baseline handgrip strength, baseline Charlson comorbidity score, visit and site as fixed factors, and subjects as an intercept only random factor. An initial test for treatment interaction was planned, and if no evidence of a significant interaction was found, the main analyses were prespecified to be conducted as two separate comparisons (perindopril vs. perindopril placebo and leucine vs. leucine placebo). The primary outcome (SPPB) was normally distributed as expected. Prespecified subgroup analyses for the primary outcome were conducted for the following categories: age ≤ 80 years versus >80 years, male versus female, and above versus below median total protein intake, together with an exploratory post hoc analysis comparing those who did and did not meet the EWGSOP2 2019 definition for confirmed sarcopenia.^18^ Although the 2010 EWGSOP criteria were used when designing the LACE trial, the 2019 criteria are the ones currently used in clinical practice and a subgroup analysis based on these updated criteria is likely to be more relevant to current clinical practice. A pre‐planned sensitivity analysis was conducted imputing the worst possible value (zero) for missing SPPB data to test the robustness of the main result. Secondary outcomes were analysed using repeated‐measures models as above, adjusted for baseline value of the variable under test, age, sex, and minimization variables.

### Meta‐analysis

To place the results of the perindopril analysis in context, we added the results of the LACE trial to our recent meta‐analysis of the effects of ACEi and ARBs on physical performance in older people.^13^ Data were analysed in RevMan v5.3 (Cochrane Collaboration). Where standard deviations (SD) of change were not available, SDs were interpolated as the mean of baseline and follow‐up SDs. Results are presented as mean differences with random‐effects meta‐analysis. To place the results of the leucine analysis in context, we used data from a recently published systematic review examining the impact of leucine on physical function.^17^ Of the 13 randomized controlled trials included in this review, three examined the effect of leucine alone as an intervention (without additional protein, amino acids, or other nutritional components). We extracted data from these three trials together with one additional trial published since^32^ and combined these data with results from the LACE trial. Data were analysed in RevMan v5.3 as described above; changes in muscle mass were presented as standardized mean differences given the heterogeneity in how results were reported.

## Results

**Figure 1:**
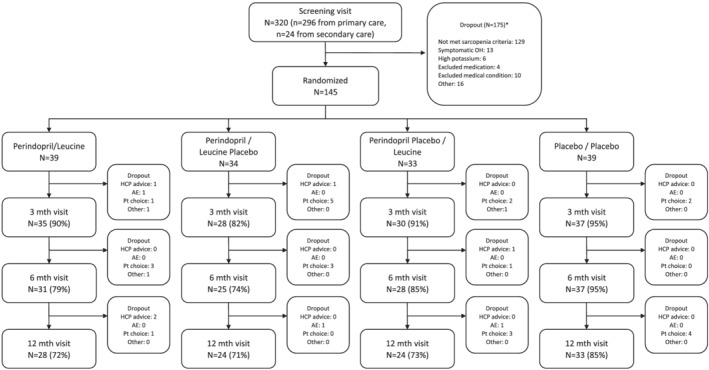
CONSORT flow diagram. AE, adverse event; HCP, health care professional; OH, orthostatic hypotension; Pt, participant.

A total of 320 participants attended a screening visit, and 145 participants were randomized into the trial between June 2016 and December 2018. The original target for recruitment was 440 participants; the trial was stopped by the funder due to slow recruitment. Figure 1 shows the flow of participants through the trial. Discontinuation of follow up was higher in the perindopril groups than in those not receiving perindopril, particularly in the first 6 months of the trial. Perindopril versus no perindopril groups and leucine versus no leucine groups were well matched at baseline for key measurements; details are given in Table 1. Adherence to intervention was lower in the group allocated to perindopril [median 76.2%, interquartile (IQR) 15.6–95.4%] than in the group not allocated to perindopril (95.9%, IQR 78.2–99.7%) (*P* < 0.001). For leucine, adherence to intervention was the same in the group allocated to leucine (median 76.2%, IQR 38.5–97.3%) as in the group not allocated to leucine (75.6%, IQR 50.5–92.3%) (*P* = 0.99).

**Table 1:** Baseline description of treatment groups

		By randomized group				By analysis group			
		Perindopril and leucine (*n* = 39)	Perindopril and leucine placebo (*n* = 34)	Perindopril placebo and leucine (*n* = 33)	Double placebo (*n* = 39)	Perindopril (*n* = 73)	No perindopril (*n* = 72)	Leucine (*n* = 72)	No leucine (*n* = 73)
Mean age (years) (SD)		78.1 (5.5)	79.5 (6.6)	78.6 (6.5)	79.0 (5.7)	78.7 (6.0)	78.8 (6.1)	78.3 (5.9)	79.3 (6.1)
Female sex (%)		22 (56)	17 (50)	16 (48)	23 (59)	39 (53)	39 (54)	38 (53)	40 (55)
Mean Charlson comorbidity score (SD)		0.8 (1.0)	0.6 (1.1)	0.6 (0.9)	0.7 (0.9)	0.7 (1.1)	0.7 (0.9)	0.7 (1.0)	0.7 (1.0)
Mean SARC‐F score (SD)		4.2 (1.3)	3.9 (1.4)	4.1 (1.3)	4.6 (1.7)	4.1 (1.3)	4.4 (1.5)	4.1 (1.3)	4.3 (1.6)
Comorbid disease									
Hypertension (%)		12 (31)	11 (32)	13 (39)	7 (18)	23 (32)	20 (28)	25 (35)	18 (25)
Ischaemic heart disease (%)		4 (10)	5 (15)	1 (3)	4 (10)	9 (12)	5 (7)	5 (7)	9 (12)
Osteoarthritis (%)		21 (54)	18 (53)	15 (45)	23 (59)	39 (53)	38 (53)	36 (50)	41 (56)
Rheumatoid arthritis (%)		1 (3)	2 (6)	5 (15)	2 (5)	3 (4)	7 (10)	6 (8)	4 (5)
Cataracts (%)		20 (51)	14 (41)	13 (39)	15 (38)	34 (47)	28 (39)	33 (46)	29 (40)
Retinopathy (%)		2 (5)	1 (3)	1 (3)	1 (3)	3 (4)	2 (3)	3 (4)	2 (3)
Registered blind (%)		1 (3)	1 (3)	0 (0)	0 (0)	2 (3)	0 (0)	1 (1)	1 (1)
Anaemia (%)		10 (26)	5 (15)	4 (12)	4 (11)	15 (21)	8 (11)	14 (19)	9 (12)
Peripheral neuropathy (%)		4 (10)	3 (9)	3 (9)	2 (5)	7 (10)	5 (7)	7 (10)	5 (7)
Previous Fragility fracture (%)		11 (28)	12 (35)	6 (18)	12 (31)	23 (32)	18 (25)	17 (24)	24 (33)
Median number of medications [IQR]		4 [2, 6]	5 [4, 7]	5 [2.5,7]	5 [3, 7]	5 [3, 7]	5 [3, 7]	5 [2, 7]	5 [3, 7]
Mean weight (kg) (SD)	Men	80.4 (12.9)	81.6 (10.1)	80.3 (9.8)	81.5 (21.1)	81.0 (11.4)	80.9 (16.0)	80.4 (11.3)	81.6 (16.1)
	Women	66.4 (12.3)	63.4 (14.2)	66.1 (7.9)	64.1 (11.3)	65.1 (13.1)	64.9 (10.0)	66.3 (10.5)	63.8 (12.5)
Mean body mass index (kg/m^2^) (SD)		27.1 (3.8)	26.2 (3.5)	27.2 (2.8)	26.8 (5.2)	26.7 (3.7)	27.0 (4.3)	27.1 (3.3)	26.5 (4.5)
Mean estimated GFR (mL/min/1.73 m^2^) (SD)		80 (21)	81 (21)	80 (18)	82 (22)	80 (20)	81 (21)	80 (19)	82 (21)
Mean serum albumin (g/L) (SD)		40 (4)	40 (4)	41 (4)	40 (4)	40 (4)	40 (4)	40 (4)	40 (4)
Mean systolic blood pressure (mmHg) (SD)		146 (20)	144 (20)	143 (18)	144 (16)	145 (20)	144 (17)	145 (19)	144 (18)
Mean diastolic blood pressure (mmHg) (SD)		80 (10)	77 (10)	77 (10)	77 (11)	79 (10)	77 (10)	79 (10)	77 (10)
Mean SPPB (SD)		7.3 (2.1)	6.8 (2.5)	6.7 (2.2)	7.1 (2.6)	7.1 (2.3)	6.9 (2.4)	7.0 (2.1)	7.0 (2.5)
Mean appendicular muscle mass by DXA (kg/m^2^) (SD)	Men	7.34 (0.84)	7.19 (0.67)	7.07 (2.02)	7.34 (1.06)	7.26 (0.75)	7.21 (1.53)	7.26 (0.87)	7.21 (1.61)
	Women	5.69 (0.58)	5.88 (0.81)	5.82 (0.56)	5.60 (0.72)	5.77 (0.68)	5.75 (0.57)	5.72 (0.76)	5.69 (0.66)
Mean maximal handgrip strength (kg) (SD)	Men	21.8 (6.7)	24.1 (5.8)	22.8 (6.4)	23.6 (4.9)	23.0 (6.3)	22.3 (6.4)	23.9 (5.3)	23.2 (5.6)
	Women	14.1 (3.6)	14.6 (4.4)	13.2 (4.3)	13.1 (3.5)	14.3 (3.9)	13.7 (3.9)	13.7 (3.9)	13.1 (3.8)
Mean maximal quadriceps strength (kg) (SD)	Men	15.0 (8.0)	15.4 (5.6)	18.8 (8.8)	15.6 (5.6)	15.2 (6.9)	16.8 (8.5)	15.5 (5.5)	17.2 (7.4)
	Women	9.7 (4.9)	11.5 (5.4)	9.6 (3.7)	11.4 (5.3)	10.5 (5.1)	9.7 (4.4)	11.5 (5.2)	10.6 (4.7)
Mean 6‐min walk distance (m) (SD)		307 (104)	288 (115)	315 (120)	311 (105)	298 (109)	313 (111)	310 (111)	301 (110)
Mean 4‐m walk speed (m/s) (SD)		0.73 (0.21)	0.73 (0.22)	0.75 (0.25)	0.77 (0.26)	0.73 (0.21)	0.76 (0.25)	0.74 (0.23)	0.75 (0.24)
Median chair rise time (s) [IQR]		21.2 [17.0, 26.8]	23.7 [18.1, 27.6]	21.9 [17.5, 27.7]	20.9 [15.4, 28.7]	22.0 [18.0, 27.4]	21.9 [16.9, 27.9]	21.6 [17.5, 26.9]	22.9 [16.3, 27.9]
Mean EQ 5D‐3L (SD)		0.77 (0.11)	0.78 (0.11)	0.78 (0.11)	0.77 (0.09)	0.77 (0.11)	0.77 (0.10)	0.77 (0.11)	0.78 (0.10)
Mean EQ 5D thermometer (SD)		71 (19)	67 (14)	76 (14)	73 (12)	69 (17)	74 (13)	73 (17)	70 (13)
Mean Nottingham EADL (SD)		56.0 (7.9)	54.6 (10.2)	54.5 (11.9)	54.2 (10.5)	55.3 (9.0)	54.3 (11.1)	55.3 (9.9)	54.4 (10.3)
Mean *T* score at hip (SD)		−1.2 (1.5)	−1.4 (1.3)	−1.2 (1.3)	−1.5 (0.9)	−1.3 (1.4)	−1.4 (1.1)	−1.2 (1.4)	−1.4 (1.1)
Mean total protein intake per day (g/kg body weight) (SD)		1.11 (0.60)	1.37 (1.36)	1.01 (0.33)	1.12 (0.33)	1.23 (1.01)	1.07 (0.33)	1.05 (0.50)	1.24 (0.96)

DXA, dual energy X‐ray absorptiometry; EADL, extended activities of daily living; EQ 5D, EuroQoL five‐dimension quality of life tool; GFR, glomerular filtration rate; SPPB, short physical performance battery.

### Primary outcome

**Figure 2:**
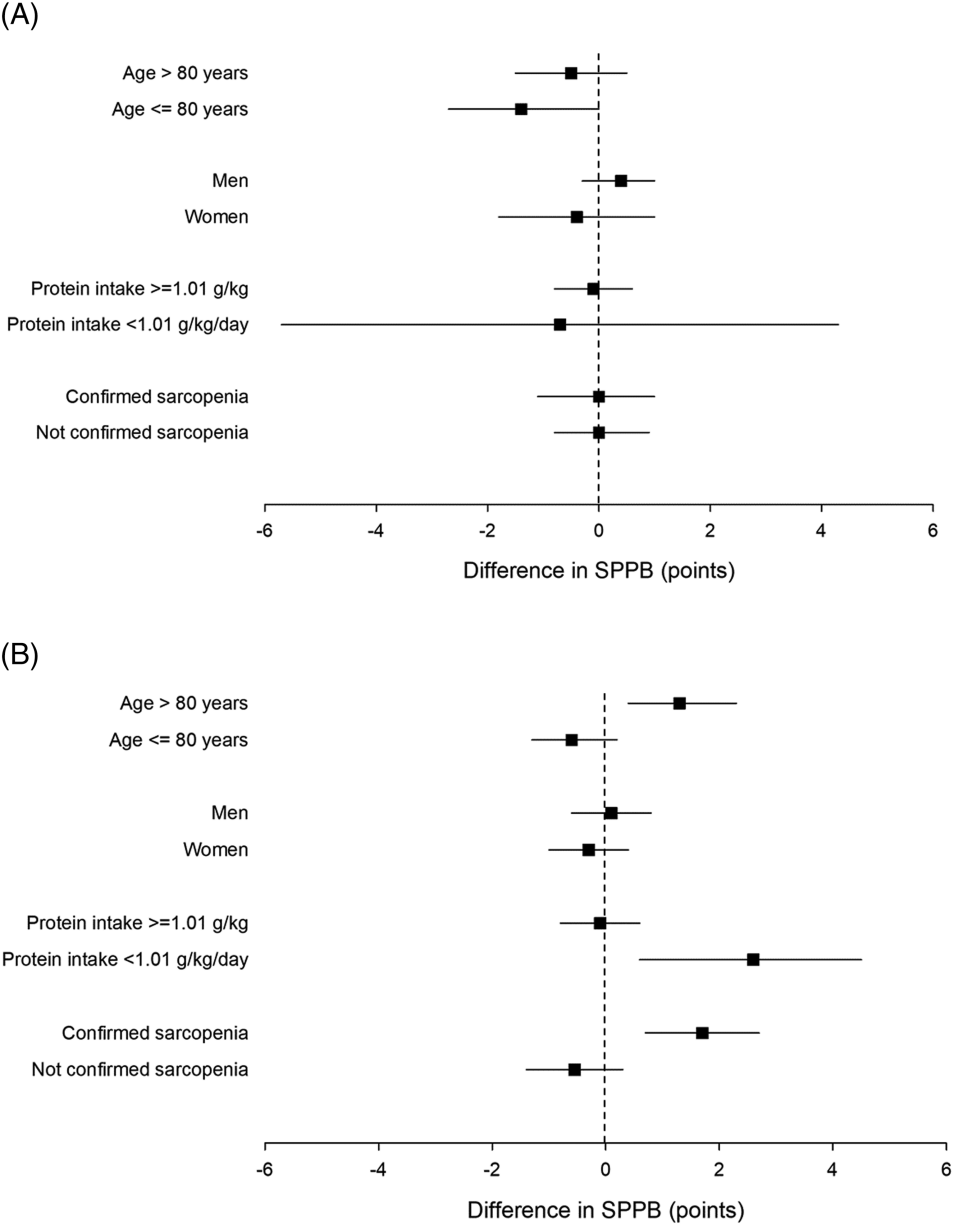
Subgroup analyses for primary outcome. (A) Perindopril versus placebo. (B) Leucine versus placebo. Positive values of short physical performance battery (SPPB) denote improvement relative to control group.

An initial test for interaction did not find evidence for an interaction between the two treatments (*P* = 0.59), and the analyses of perindopril versus no perindopril and leucine versus no leucine were therefore conducted separately as planned. Tables 2 and 3 show the analyses for the primary outcome (between‐group difference in SPPB) for perindopril versus no perindopril and for leucine versus no leucine, respectively. No significant treatment effect was seen in unadjusted or adjusted analyses; the point estimate of effect in adjusted analyses was close to zero although the confidence intervals do not exclude an effect size consistent with a clinically important difference of 1.0 point. Sensitivity analyses examining the difference at the 12‐month timepoint, and imputing values of zero as a worst‐case scenario showed similar results. Subgroup analyses are shown in Figure 2. For the leucine comparison, participants with protein intake below the median level of 1.01 g/kg/day showed a treatment effect of 2.6 points (95%CI 0.6 to 4.5) compared with −0.1 points (95%CI −0.8 to 0.6) for those with a protein intake above the median; however, the subgroup interaction was not significant on formal analysis (*P* = 0.70). Similarly, participants meeting the full EWGSOP 2019 criteria for sarcopenia (*n* = 44) showed a greater leucine treatment effect (1.7 points, 95%CI 0.7 to 2.7) compared with those not meeting the criteria (−0.5 points, 95%CI −1.4 to 0.3); *P* for interaction = 0.06; those aged over 80 also showed a slightly greater leucine treatment effect (1.3 points, 95%CI 0.4 to 2.3 vs. −0.7 points, 95%CI −1.3 to 0.2 points; *P* for interaction = 0.76). Adherence did not have a significant interaction with the primary outcome when included in the adjusted models as a continuous variable (perindopril model interaction: *P* = 0.75; leucine model interaction: *P* = 0.85).

**Table 2:** Primary outcome in those randomized to perindopril versus perindopril placebo

	Perindopril (*n* = 73)	No perindopril (*n* = 72)	Unadjusted treatment effect [95% CI]	*P*	Adjusted treatment effect [95% CI]	*P*
Baseline SPPB (N, SD)	7.1 (73, 2.3)	6.9 (72, 2.4)	0.0 [−0.7, 0.8]	0.91	−0.1 [−1.2, 1.0]	0.89
6‐month SPPB (N, SD)	7.3 (56, 2.5)	7.0 (65, 2.7)				
12‐month SPPB (N, SD)	7.2 (52, 2.9)	7.6 (57, 2.6)				
Sensitivity analyses (12 months only)						
12‐month SPPB (N, SD)	7.2 (52, 2.9)	7.6 (57, 2.6)	−0.6 [−1.4, 0.2]	0.12	0.5 [−2.6, 3.6]	0.73
12‐month SPPB, worst‐case^a^ (N, SD)	5.1 (73. 4.1)	6.0 (72, 3.9)	−1.0 [−2.2, 0.2]	0.10	0.2 [−2.4, 2.8]	0.87

SPPB, short physical performance battery.

Adjusted analyses adjusted for age, sex, SPPB, Charlson comorbidity score, and baseline handgrip strength.

^a^ Imputing SPPB = 0 for all missing data.

**Table 3:** Primary outcome in those randomized to leucine versus leucine placebo

	Leucine (*n* = 72)	No leucine (*n* = 73)	Unadjusted treatment effect [95% CI]	*P*	Adjusted treatment effect [95% CI]	*P*
Baseline SPPB (N, SD)	7.0 (72, 2.1)	7.0 (73, 2.5)	0.1 [−0.7, 0.8]	0.83	0.1 [−1.0, 1.1]	0.90
6‐month SPPB (N, SD)	7.2 (59, 2.6)	7.1 (62, 2.6)				
12‐month SPPB (N, SD)	7.3 (52, 2.7)	7.5 (57, 2.8)				
Sensitivity analysis (12 months only)						
12‐month SPPB (N, SD)	7.3 (52, 2.7)	7.5 (57, 2.8)	0.0 [−0.8, 0.8]	0.98	−0.5 [−3.1, 2.0]	0.66
12‐month SPPB, worst‐case^a^ (N, SD)	5.3 (72, 4.0)	5.8 (73, 4.0)	−0.6 [−1.8, 0.6]	0.34	0.6 [−1.9, 3.1]	0.60

SPPB, short physical performance battery.

Adjusted analyses adjusted for age, sex, SPPB, Charlson comorbidity score and baseline handgrip strength.

^a^ Imputing SPPB = 0 for all missing data.

### Secondary outcomes

Adjusted treatment effects for the secondary outcomes are shown in Table 4. Perindopril treatment was associated with a worse health status than placebo on the EQ 5D thermometer tool [−12 points (95% CI −21 to −3); *P* = 0.01], and leucine treatment was associated with worse scores on the main EQ 5D health status tool [−0.06 points (95% CI −0.11 to −0.01); *P* = 0.03]. No other significant treatment effects were found.

**Table 4:** Secondary outcomes in those randomized to perindopril versus perindopril placebo and leucine versus leucine placebo

	Perindopril versus no perindopril					Leucine versus no leucine				
Outcome	Timepoint	Perindopril	Placebo	Adjusted treatment effect [95% CI]	*P*	Timepoint	Leucine	Placebo	Adjusted treatment effect [95% CI]	*P*
Muscle mass	Baseline (*n*, SD)	6.47 (73, 1.03)	6.38 (72, 1.41)	−0.4 [−1.1, 0.3]	0.27	Baseline (*n*, SD)	6.44 (72, 1.34)	6.42 (73, 1.12)	−0.3 [−1.0, 0.4]	0.47
	12 months (*n*, SD)	6.09 (54, 2.18)	6.22 (58, 1.89)			12 months (*n*, SD)	6.03 (54, 2.23)	6.28 (58, 1.83)		
Grip strength	Baseline (*n*, SD)	18.3 (73, 6.7)	17.8 (72, 6.9)	0.2 [−0.9, 1.2]	0.74	Baseline (*n*, SD)	17.8 (72, 6.8)	18.3 (73, 6.8)	−0.3 [−1.2, 0.7]	0.55
	6 months (*n*, SD)	19.9 (56, 7.0)	18.0 (65, 7.5)			6 months (*n*, SD)	18.8 (59, 7.3)	19.0 (62, 7.5)		
	12 months (*n*, SD)	20.4 (50, 6.7)	19.0 (55, 6.9)			12 months (*n*, SD)	19.4 (50, 6.5)	19.9 (55, 7.1)		
Quadriceps strength (kg)	Baseline (*n*, SD)	12.7 (67, 6.4)	13.53 (68, 6.8)	0.6 [−3.0, 4.1]	0.75	Baseline (*n*, SD)	12.9 (68, 7.4)	13.3 (67, 5.7)	−1.0 [−4.4, 2.4]	0.55
	6 months (*n*, SD)	14.5 (53, 8.3)	12.40 (58, 5.4)			6 months (*n*, SD)	12.6 (56, 5.6)	14.2 (55, 8.2)		
	12 months (*n*, SD)	13.6 (40, 6.2)	14.36 (48, 7.3)			12 months (n, SD)	13.9 (44, 7.0)	14.2 (44, 6.6)		
Six min walk (m)	Baseline (*n*, SD)	298 (73, 109)	313 (71, 111)	−32 [−75, 12]	0.15	Baseline (*n*, SD)	311 (71, 111)	301 (73, 110)	17 [−25, 59]	0.43
	6 months (*n*, SD)	328 (54, 103)	321 (60, 111)			6 months (*n*, SD)	330 (57, 98)	318 (57, 116)		
	12 months (*n*, SD)	338 (46, 97)	324 (54, 115)			12 months (*n*, SD)	328 (49, 103)	333 (51, 112)		
Gait speed (m/s)	Baseline (*n*, SD)	0.73 (73, 0.21)	0.76 (72, 0.25)	0.01 [−0.18, 0.19]	0.96	Baseline (*n*, SD)	0.74 (72, 0.23)	0.75 (73, 0.24)	0.01 [−0.18, 0.19]	0.96
	6 months (*n*, SD)	0.85 (55, 0.28)	0.86 (61, 0.43)			6 months (*n*, SD)	0.81 (57, 0.30)	0.90 (59, 0.42)		
	12 months (*n*, SD)	0.84 (49, 0.25)	1.00 (56, 1.11)			12 months (*n*, SD)	0.85 (50, 0.27)	1.00 (55, 1.12)		
Chair stand time (s)	Baseline (*n*, SD)	24.1 (58, 9.3)	24.2 (53, 11.8)	−1.7 [−8.7, 5.3]	0.64	Baseline (*n*, SD)	24.4 (54, 9.3)	23.8 (54, 9.3)	−3.1 [−9.5, 3.3]	0.34
	6 months (*n*, SD)	22.1 (44, 9.0)	22.5 (51, 10.3)			6 months (*n*, SD)	21.7 (47, 11.0)	23.0 (48, 8.2)		
	12 months (*n*, SD)	21.3 (38, 12.9)	22.4 (47, 10.9)			12 months (*n*, SD)	22.6 (40, 14.8)	21.3 (45, 8.4)		
NEADL	Baseline (*n*, SD)	55.3 (73, 9.0)	54.3 (72, 11.1)	−1.6 [−7.4, 4.2]	0.58	Baseline (*n*, SD)	55.3 (72, 9.9)	54.4 (73, 10.3)	−2.0 [−7.4, 3.5]	0.48
	6 months (*n*, SD)	56.6 (56, 8.0)	54.3 (65, 11.2)			6 months (*n*, SD)	55.9 (59, 9.0)	54.9 (62, 10.7)		
	12 months (*n*, SD)	56.2 (51, 10.6)	55.3 (55, 10.5)			12 months (*n*, SD)	56.0 (50, 9.2)	55.4 (56, 11.6)		
EQ 5D main score	Baseline (*n*, SD)	0.77 (70, 0.11)	0.77 (70, 0.10)	−0.04 [−0.10, 0.02]	0.23	Baseline (*n*, SD)	0.77 (67, 0.11)	0.78 (73, 0.10)	−0.06 [−0.11, −0.01]	0.03
	6 months (*n*, SD)	0.79 (53, 0.11)	0.82 (64, 0.13)			6 months (*n*, SD)	0.80 (56, 0.13)	0.81 (61, 0.11)		
	12 months (*n*, SD)	0.77 (50, 0.10)	0.81 (56, 0.13)			12 months (*n*, SD)	0.81 (50, 0.13)	0.77 (56, 0.10)		
EQ 5D thermometer	Baseline (*n*, SD)	69 (71, 17)	74 (71, 13)	−12 [‐3, ‐21]	0.01	Baseline (*n*, SD)	73 (70, 17)	70 (72, 13)	‐3 [−12, 6]	0.53
	6 months (*n*, SD)	67 (55, 18)	74 (65, 15)			6 months (*n*, SD)	72 (59, 18)	69 (61, 16)		
	12 months (*n*, SD)	69 (51, 18)	75 (56, 14)			12 months (*n*, SD)	72 (51, 20)	72 (56, 13)		
*T* score at femoral neck	Baseline (*n*, SD)	−1.29 (64, 1.41)	−1.36 (64, 1.06)	0.03 [−0.74, 0.81]	0.93	Baseline (*n*, SD)	−1.22 (63, 1.35)	−1.42 (65, 1.10)	0.17 [−0.59, 0.93]	0.66
	12 months (*n*, SD)	−1.00 (42, 1.31)	−1.40 (50, 1.05)			12 months (*n*, SD)	−1.14 (44, 1.35)	−1.29 (48, 1.01)		
Median HOMA‐IR	Baseline [*n*, IQR]	2.9 [65, 2.2–4.1]	2.8 [62, 2.0, 4.8]	−1.8 [−5.1, 1.4]	0.26	Baseline [*n*, IQR]	2.8 (63, 2.0–4.2)	3.1 (64, 2.2–4.8)	−1.3 [−4.5, 1.9]	0.42
	3 months [*n*, IQR]	2.9 [54, 1.9–4.9]	3.4 [53, 1.7, 7.0]			3 months [*n*, IQR]	3.0 (55, 2.0–6.8)	2.7 (52, 1.6–5.8)		
	12 months [*n*, IQR]	3.0 [49, 2.0–4.9]	2.6 [52, 1.6, 5.0]			12 months [*n*, IQR]	2.6 (49, 1.9–5.1)	3.1 (52, 1.8–4.8)		

EQ 5D, EuroQoL 5D score; HOMA‐IR, homeostatic model assessment of insulin resistance; NEADL, Nottingham extended activities of daily living.

Analyses adjusted for age, sex, SPPB, Charlson comorbidity score, and baseline handgrip strength.

### Adverse events

Table 5 shows key prespecified adverse events of interest. Hyperkalaemia (two cases in perindopril group and no cases in no perindopril group) and important rises in serum creatinine (one case in perindopril group and no cases in no perindopril group) were infrequent. Seven participants in the groups allocated to perindopril experienced hyponatremia on at least one test during the trial, compared with two in the no perindopril group. One death (due to acute leukaemia and unrelated to treatment) was noted in the perindopril and leucine placebo arm. There was no significant difference in the falls rate between those allocated to perindopril and perindopril placebo, or between those allocated to leucine and leucine placebo; rates of fragility fractures were low in all groups. Figure S2 shows changes in supine blood pressure and orthostatic reduction in blood pressure for those allocated to perindopril versus no perindopril; supine blood pressure fell in the perindopril group relative to no perindopril as expected, but little difference in the magnitude of the postural fall in blood pressure was observed between groups. Table S4 shows the full categorization of adverse events in each arm. The overall number of adverse events was higher in those receiving perindopril, driven by higher rates of injuries, nervous system disorders, and gastrointestinal disorders. Overall numbers of adverse events were similar in those allocated to leucine or leucine placebo.

**Table 5:** Key adverse outcomes of interest

	Perindopril (*n* = 73)	No perindopril (*n* = 72)	Leucine (*n* = 72)	No Leucine (*n* = 73)
Deaths (%)	1 (1)	0 (0)	0 (0)	1 (1)
Number of participants with fragility fractures^a^ (%)	3 (4)	1 (1)	1 (1)	3 (4)
Number of participants with at least one fall (%)	30 (41)	37 (51)	34 (47)	30 (41)
Number of falls	121	132	121	132
Falls per year (95% CI)	2.0 (1.1, 3.0)	2.8 (0.6, 5.1)	1.9 (0.9, 2.9)	2.9 (0.8, 5.0)

^a^ Distal radius, symptomatic vertebra, or neck of femur.

### Updated meta‐analyses

Table 6 shows the results of incorporating the LACE trial results into meta‐analyses with other trials of either ACEi/ARB or leucine to improve muscle function in older people; forest plots are shown in Figure S3 and included study details are shown in Table S5. Heterogeneity for most analyses was low. For the SPPB, the meta‐analysis results excluded the MCID of 0.5 points suggested by previous work.^29^ The results for handgrip strength and quadriceps strength similarly excluded the MCIDs for these measures^33^, ^34^; for the 6‐min walk, the 95% confidence intervals did not exclude the MCID of 20 m for older people,^29^ although the summary point estimate did not support a clinically important effect. For leucine, the wider confidence intervals did not exclude a clinically important treatment effect, but summary point estimates for all measures studied (including muscle mass) did not support a clinically important treatment effect.

**Table 6:** Summary meta‐analysis results

	ACEi/ARB				Leucine			
Outcome	*N* of studies	*N* (ACEi, control)	Treatment effect (95% CI)	*I* ^2^	*N* of studies	*N* (leucine, control)	Treatment effect (95% CI)	*I* ^2^
SPPB	4	439, 423	−0.1 (−0.4, 0.2)	0	ND	ND	ND	ND
6‐min walk distance (m)	4	239, 237	0 (−28 to 27)	75	2	80, 81	−4 (−27 to 35)	0
Walk speed (m/s)	ND	ND	ND	ND	2	95, 92	0.00 (−0.15 to 0.15)	0
Handgrip strength (kg)	4	439, 423	−0.2 (−1.0 to 0.7)	0	3	103, 100	−0.4 (−2.4, 1.5)	0
Quadriceps strength (kg)	5	270, 251	−0.8 (−1.9 to 0.3)	0	2	101, 101	−0.7 (−2.6 to 1.3)	0
Muscle mass (*z* score)	ND	ND	ND	ND	5	147, 142	−0.04 (−0.27 to 0.19)	0

ACEi, angiotensin converting enzyme inhibitor; ARB, angiotensin receptor blocker; ND, Insufficient data to combine in meta‐analysis; SPPB, short physical performance battery.

## Discussion

We found no evidence that perindopril or leucine improved physical performance, muscle mass, or quality of life in older people with sarcopenia and the evidence from meta‐analysis of the LACE trial and other trials of ACEi/ARBs or leucine does not support a clinically meaningful improvement in physical performance with these agents. This was despite adequate adherence to both perindopril and leucine. Although no excess of adverse events was noted with leucine use, perindopril was associated with a higher rate of adverse events than placebo as would be expected from the known side effects of ACEi, and participants allocated to perindopril reported a poorer quality of life than those not allocated to perindopril although this could be a chance finding due to multiple testing.

When the LACE trial was designed, preclinical and mechanistic clinical data supported potential beneficial modes of action for these agents, and for both interventions, some clinical trial data suggesting efficacy also existed.^15^, ^16^, ^31^ There are several reasons for the lack of efficacy seen in the LACE trial, which merit consideration. Firstly, it is possible that the intervention dose or duration were not adequate to provide a clinically important response. However, the perindopril dose used has previously demonstrated improvement in 6‐min walk distance and quality of life in older people with functional impairment.^31^ Recent observational data suggest that ARB use, but not ACEi use, is associated with better muscle strength and muscle mass,^35^ but ARBs have not improved muscle function in trials to date.^36^, ^37^ The leucine dose used was sufficient to improve muscle protein synthesis in healthy older people.^15^, ^16^ It is still possible, however, that a higher dose is required to overcome anabolic resistance in older people with sarcopenia. No relationship was evident between adherence and treatment effect for the primary outcome for either perindopril or leucine.

Secondly, it is possible that ACEi or leucine is efficacious only when used in combination with resistance training for sarcopenia. However, at the time of designing the LACE trial, the existing evidence suggested that the opposite might be true for ACEi/ARB^38^, ^39^; a similar lack of efficacy was seen when adding the ARB losartan to resistance training in older people.^37^ The case for or against leucine as an adjunct to resistance training is less clear‐cut. Thirdly, it is possible that for leucine, efficacy is achievable only as part of a more complex nutritional intervention, or only in those with low baseline protein intake as suggested by our subgroup analysis. Some previous interventions have combined leucine with additional protein or amino acid supplements and with other nutrients such as vitamin D.^17^, ^40^ Some, but not all, of these trials have suggested improvements to muscle mass, although the effect on muscle strength has been less convincing. Current evidence is insufficient to indicate whether leucine is an effective intervention when given in addition to generic protein or amino acid supplementation; further trials are also required examining efficacy in those with low baseline protein intake.

The LACE trial had a number of strengths; it is one of the few trials to have been designed specifically to recruit older people with sarcopenia and is one of very few multicentre randomized controlled sarcopenia trials. Almost all participants fulfilled the EWGSOP2 2019 definition for probable sarcopenia—a definition increasingly used in clinical practice. The trial participants had high levels of comorbidity and poor physical performance and were thus representative of patients typically seen in primary care and in secondary care Older Peoples Medicine services—both key services for the detection and treatment of sarcopenia. Other important strengths include the 1 year follow up, which is longer than used in most sarcopenia trials to date, and the comprehensive set of outcome measures including both measures of physical performance and measures such as quality of life that are of importance to patients.^41^

The key limitation of the trial was that we were unable to recruit the original target population size of 440 participants as previously discussed.^42^ Although sarcopenia is common in the older population, it is commonly accompanied by multiple comorbidities that may prevent individuals from being eligible for trials. It is particularly common in care home residents (the majority of whom have dementia in the United Kingdom) and is often accompanied by mobility limitation that may prevent participation in research. In addition, we found that many potential participants had low muscle strength but still had preserved muscle mass, making them ineligible for the trial. The reduced sample size raises the possibility that the null result was due to insufficient statistical power. This sample size was calculated using a conservative 0.5 point difference in the SPPB as the MCID. The original sample size calculation was also conservative in that it did not factor in the increased statistical power inherent in the repeated‐measures analysis. Nevertheless, the sample size and consequently the 95% confidence intervals were not sufficiently narrow to exclude the MCID of 0.5 points in the SPPB. Meta‐analysis of trials combining the LACE results with other trials did however exclude this MCID. We did not undertake adjustment for multiple testing, and had we done so, it is likely that the worsening in quality of life scores we observed with both perindopril and leucine would have not reached significance.

Adherence to perindopril was lower than adherence to placebo, in part because of side effects prompting discontinuation of treatment. Although this is likely to have diluted the treatment effect, the adherence to perindopril is higher than would be expected in clinical practice when perindopril is used as an antihypertensive; adherence to antihypertensives is poor with discontinuation rates of 50% or more.^43^ Our results are likely therefore to give a realistic estimate of what might be achievable in routine clinical practice if ACEi were to be used as agents to treat sarcopenia. Similarly, although leucine adherence was not optimal, it is unlikely that perfect adherence could be achieved in clinical practice. Although we selected a dose of 2.5 g three times a day for leucine, this was based on muscle protein synthesis studies in healthy older people, and it is also possible that higher doses of leucine are required in people with sarcopenia to overcome anabolic resistance effectively in this group.

The generalizability of our results is limited by the fact that participants were overwhelmingly of white ethnicity, and thus, the results cannot be assumed to apply to other ethnicities. Despite our efforts to enrol patients meeting the EWGSOP 2010 guidelines, fewer than half of participants fully met the criteria for confirmed sarcopenia under the 2010 guidelines. This was in large part due to our use of bioimpedance measures for muscle mass screening and confirmation of eligibility to enter the trial. Some individuals with low muscle mass on bioimpedance testing had preserved muscle mass on the dual energy X‐ray absorptiometry measurement that was used to categorize participants as meeting the full EWGSOP sarcopenia definition. Measuring muscle mass at scale remains a challenge for sarcopenia trials, and the bioimpedance measurement process we used was not able to identify those muscle mass below the sarcopenia diagnostic threshold with high accuracy. However, the guidelines have changed since the trial was designed, and almost all participants met the criteria for probable sarcopenia under the EWGSOP2 2019 guidelines. It is still possible as discussed above that the treatment effect in patients with lower muscle mass (confirmed sarcopenia) may be greater than those seen in patients who do not fulfil the criteria for confirmed sarcopenia.

In conclusion, our results do not suggest sufficient evidence to support the use of either perindopril or leucine as standalone interventions to improve physical performance or muscle mass in older people with sarcopenia. Although current evidence does not support further trials of ACE inhibitors as standalone or adjuncts to exercise for people with sarcopenia, further trials are needed to test whether leucine could benefit patient subgroups with low muscle mass and/or low protein intake.

## Funding

The LACE trial (project reference 13/53/03) is funded by the Efficacy and Mechanism Evaluation (EME) Programme, an MRC and NIHR partnership. The views expressed in this publication are those of the authors and not necessarily those of the MRC, NIHR or the Department of Health and Social Care.

## Conflict of interests

Professor Donnan is a member of the New Drugs Committee of the Scottish Medicines Consortium and has received recent grants from AbbVie Pharmaceuticals. All other authors declare no competing interests.

## Ethical statement

All authors of this manuscript comply with the guidelines of ethical authorship and publishing in the *Journal of Cachexia, Sarcopenia and Muscle*.^44^

## Supporting information


**Table S1:** Body mass index and sex‐specific screening cut offs for bioimpedance derived appendicular muscle mass
Table S2: Exclusion criteria
Table S3: List of all secondary outcomes
Table S4. Adverse events by System Order Class
Table S5: Trials included in meta‐analysis
Figure S1: Flowchart for perindopril uptitration
Figure S2: Change in lying blood pressure and postural blood pressure drop for perindopril vs placebo analysis
Figure S3. Meta‐analysis Forest plots

## Appendix A The LACE study group members

**Table:**

Name	Affiliation
Marcus Achison^1^	Tayside Clinical Trials Unit (TCTU), Tayside Medical Science Centre (TASC), University of Dundee, Ninewells Hospital & Medical School, Dundee, UK
Simon Adamson^1^	Tayside Clinical Trials Unit (TCTU), Tayside Medical Science Centre (TASC), University of Dundee, Ninewells Hospital & Medical School, Dundee, UK
Asangaedem Akpan^2^	Clinical Research Network Northwest Coast, University of Liverpool, Liverpool University Hospitals NHS FT Trust, Liverpool, UK
Terry Aspray^3^	AGE Research Group, NIHR Newcastle Biomedical Research Centre, Translational Clinical Research Institute, Newcastle University and Newcastle‐upon‐Tyne NHS Trust, Newcastle upon Tyne, UK
Alison Avenell^4^	Health Services Research Unit, University of Aberdeen, Aberdeen, UK
Margaret M. Band^1^	Tayside Clinical Trials Unit (TCTU), Tayside Medical Science Centre (TASC), University of Dundee, Ninewells Hospital & Medical School, Dundee, UK
Tufail Bashir^5^	Cardiovascular and Respiratory Interface Section, National Heart and Lung Institute, Imperial College London, South Kensington Campus, London, UK
Louise A. Burton^6^	Medicine for the Elderly, NHS Tayside, Dundee, UK and Ageing and Health, University of Dundee, Dundee, UK
Vera Cvoro^7,8^	Victoria Hospital, Kirkcaldy, UK and Centre for Clinical Brain Sciences University of Edinburgh, Edinburgh, UK
Peter T. Donnan^9^	Division of Population Health and Genomics, School of Medicine, University of Dundee, Dundee, UK
Gordon W. Duncan^10^	Medicine for the Elderly, NHS Lothian, Edinburgh, UK and Centre for Clinical Brain Sciences, University of Edinburgh, Edinburgh, UK
Jacob George^11^	Department Clinical Pharmacology, Division of Molecular & Clinical Medicine, University of Dundee Medical School, Ninewells Hospital, Dundee, UK
Adam L. Gordon^12,13,14^	Unit of Injury, Inflammation and Recovery, School of Medicine, University of Nottingham; NIHR Nottingham Biomedical Research Centre, Nottingham, UK; Department of Medicine for the Elderly, University Hospitals of Derby and Burton NHS Foundation Trust, Derby, UK.
Celia L. Gregson^15,16^	Musculoskeletal Research Unit, Bristol Medical School, University of Bristol, Bristol, UK; Older Person's Unit, Royal United Hospital NHS Foundation Trust Bath, Combe Park, Bath, UK
Adrian Hapca^1^	Tayside Clinical Trials Unit (TCTU), Tayside Medical Science Centre (TASC), University of Dundee, Ninewells Hospital & Medical School, Dundee, UK
Emily Henderson^17,18^	Population Health Sciences, Bristol Medical School, University of Bristol, Bristol, UK; Royal United Hospital Bath NHS Foundation Trust, Bath, UK.
Cheryl Hume^1^	Tayside Clinical Trials Unit (TCTU), Tayside Medical Science Centre (TASC), University of Dundee, Ninewells Hospital & Medical School, Dundee, UK
Thomas A. Jackson^19^	Institute of Inflammation and Ageing, University of Birmingham, Birmingham, UK
Paul Kemp^20^	Cardiovascular and Respiratory Interface Section, National Heart and Lung Institute, Imperial College London, South Kensington Campus, London, UK
Simon Kerr^20^	Department of Older People's Medicine, Newcastle upon Tyne Hospitals NHS Foundation Trust, Newcastle upon Tyne
Alixe Kilgour^21^	Medicine for the Elderly, NHS Lothian, Edinburgh, UK
Veronica Lyell^1^	Older Person's Unit, Royal United Hospital NHS Foundation Trust Bath, Combe Park, Bath, UK
Tahir Masud^22^	Clinical Gerontology Research Unit, Nottingham University Hospitals NHS Trust, City Hospital Campus, Nottingham, UK
Andrew McKenzie^1^	Tayside Clinical Trials Unit (TCTU), Tayside Medical Science Centre (TASC), University of Dundee, Ninewells Hospital & Medical School, Dundee, UK
Emma McKenzie^1^	Tayside Clinical Trials Unit (TCTU), Tayside Medical Science Centre (TASC), University of Dundee, Ninewells Hospital & Medical School, Dundee, UK
Harnish Patel^23^	NIHR Biomedical Research Centre, University of Southampton and University Hospital Southampton NHSFT, Southampton, UK
Kristina Pilvynte^1^	Tayside Clinical Trials Unit (TCTU), Tayside Medical Science Centre (TASC), University of Dundee, Ninewells Hospital & Medical School, Dundee, UK
Helen C. Roberts^24^	Academic Geriatric Medicine, University of Southampton, Mailpoint 807 Southampton General Hospital, Southampton, UK
Christos Rossios^5^	Cardiovascular and Respiratory Interface Section, National Heart and Lung Institute, Imperial College London, South Kensington Campus, London, UK
Avan A. Sayer^3^	AGE Research Group, NIHR Newcastle Biomedical Research Centre, Translational Clinical Research Institute, Newcastle University and Newcastle‐upon‐Tyne NHS Trust, Newcastle upon Tyne, UK
Karen T. Smith^1^	Tayside Medical Science Centre (TASC), University of Dundee, Ninewells Hospital & Medical School, Dundee, UK
Roy L. Soiza^25^	Ageing & Clinical Experimental Research (ACER) Group, University of Aberdeen, Aberdeen, UK
Claire J. Steves^26^	Department of Twin Research and Genetic Epidemiology, King's College London & Department of Clinical Gerontology, King's College Hospital, London, UK.
Allan D. Struthers^27^	Division of Molecular and Clinical Medicine, University of Dundee, Ninewells Hospital, Dundee, UK
Deepa Sumukadas^28^	Department of Medicine for the Elderly, NHS Tayside, Dundee, UK
Divya Tiwari^29^	Bournemouth University and Royal Bournemouth Hospital, Bournemouth, UK.
Julie Whitney^30^	School of Population Health & Environmental Sciences, King's College London and King's College Hospital, London, UK
Miles D. Witham^3^	AGE Research Group, NIHR Newcastle Biomedical Research Centre, Translational Clinical Research Institute, Newcastle University and Newcastle‐upon‐Tyne NHS Trust, Newcastle upon Tyne, UK
