# Supplementary material for: Effect of perindopril or leucine on physical performance in older people with sarcopenia: the LACE randomized controlled trial
Source: J Cachexia Sarcopenia Muscle. 2022 Feb 16;13(2):858–71. doi: 10.1002/jcsm.12934 (PMC8977979; doi:10.1002/jcsm.12934)
Supplement: Supplementary file 1 — Table S1: Body mass index and sex‐specific screening cut offs for bioimpedance derived appendicular muscle mass Table S2: Exclusion criteria Table S3: List of all secondary outcomes Table S4. Adverse events by System Order Class Table S5: Trials included in meta‐analysis Figure S1: Flowchart for perindopril uptitration Figure S2: Change in lying blood pressure and postural blood pressure drop for perindopril vs placebo analysis Figure S3. Meta‐analysis Forest plots [file JCSM-13-858-s001.pdf]

Effect of Perindopril or Leucine on physical performance in older people with sarcopenia: the LACE randomised controlled trial

Supplementary information

Supplementary Table 1: Body mass index and sex-specific screening cut offs for bioimpedance derived appendicular muscle mass:

	Muscle mass cutoffs	
	Men	Women
Body mass index <18.5 kg/m^2	<=6.02 kg/m^2	<=5.25 kg/m^2
18.5 – 24.9	<=7.14	<=5.70
25.0 – 29.9	<=8.00	<=6.19
>=30 kg/m^2	<=8.77	<=6.72

Supplementary Table 2: Exclusion criteria

a) *Contraindications or existing indications to therapies or placebo*
-Known clinical diagnosis of chronic heart failure (by European Society of Cardiology criteria)
-Confirmed LV systolic dysfunction on any imaging modality
-Known aortic stenosis (peak gradient >30mmHg)
-Systolic BP<90 mmHg (supine)
-Dizziness on standing associated with a postural drop of >20/10mmHg (asymptomatic orthostatic hypotension will not be a contraindication)
-Serum Creatinine >170 umol/L or eGFR<30ml/min by MDRD4 calculation
-K>5.0 mmol/L; Na<130 mmol/L
-Using ACEi, Angiotensin receptor blocker, aldosterone blocker or leucine already
-Previous adverse reaction to ACEi or leucine
-Current use of oral NSAIDs (aspirin is permitted, as are topical NSAIDs)
-Current use of potassium supplements, aliskiren, spironolactone or other potassium-sparing diuretics
-Hereditary or idiopathic angioedema
-Lactose intolerance
b) *Contraindications to consent or undertaking study outcomes*
-Implantable cardioverter defibrillator or pacemaker with atrial sensing lead (pacemakers with ventricular sensing lead only are allowed)
-Peripheral oedema present above knee level
-Unable to mobilise without human assistance (walking aids allowed)
-Unable to give written informed consent

-Currently enrolled in another intervention research study, or less than 30 days since completing another intervention research study. Concomitant enrolment in observational studies is permitted.
c) *Overlap with other myopathic conditions or important confounders*
-Currently enrolled in a time-limited exercise-based rehabilitation programme
-Any progressive neurological or malignant condition with life expectancy <6 months
-Severe COPD (GOLD stage IV)
-Known myositis or other established myopathy
-Self-reported weight loss of >10% in last 6 months (to exclude significant cachexia)
-Known uncontrolled thyrotoxicosis
-7.5mg/day or greater prednisolone use (or equivalent)

Supplementary Table 3: List of all secondary outcomes

Outcome	Measurement details	Measurement timepoints
Maximum grip strength	Jamar dynamometer; best of three [1]	0, 6 and 12 months
Maximum Quadriceps strength	Lafayette dynamometer; best of three [2]	0, 6 and 12 months
Six-minute walk distance	25m course with standardised encouragement [3]	0, 6 and 12 months
Four metre walk speed	Done as part of Short Physical Performance Battery [4]	0, 6 and 12 months
Five times sit to stand test	Done as part of Short Physical Performance Battery [5]	0, 6 and 12 months
Instrumental Activities of Daily Living	Nottingham extended ADL questionnaire [6]	0, 6 and 12 months
Health-related quality of life	EuroQoL EQ5D-3L questionnaire [7]	0, 6 and 12 months
Appendicular muscle mass / height squared	Dual energy X-ray absorptiometry (DXA)	0 and 12 months
Neck of femur bone mineral density	Dual energy X-ray absorptiometry (DXA)	0 and 12 months
Insulin resistance	Homeostatic measure of Insulin Resistance (HOMA-IR) [8]	0, 3 and 12 months

Supplementary Table 4. Adverse events by System Order Class

	Perindopril and leucine	Perindopril and leucine placebo	Perindopril placebo and leucine	Double placebo
Number with at least one adverse event (%)	38 (97)	31 (91)	29 (88)	33 (85)
Number of adverse events:	117	101	70	95
Blood and lymphatic system disorders	0	2	2	0
Cardiac disorders	5	1	2	5
Eye disorders	2	1	2	3
Gastrointestinal disorders	20	17	12	14
General disorders and administration site conditions	3	6	2	4
Hepatobiliary disorders	0	0	1	0
Infections and infestations	20	20	14	24
Injury, poisoning and procedural complications	13	9	6	6
Investigations	2	0	1	1
Metabolism and nutrition disorders	7	1	1	2
Musculoskeletal and connective tissue disorders	12	9	8	11
Neoplasms benign, malignant and unspecified (incl cysts and polyps)	0	5	1	3
Nervous system disorders	15	11	9	8
Psychiatric disorders	1	5	1	0
Renal and urinary disorders	2	3	0	1
Reproductive system and breast disorders	0	2	0	0
Respiratory, thoracic and mediastinal disorders	6	4	3	6
Skin and subcutaneous tissue disorders	5	4	1	7
Vascular disorders	4	1	4	0

Supplementary Table 5: Trials included in meta-analysis

	Country	N	Mean age	% women	Inclusion criteria	Intervention	Comparator	Primary outcome	Secondary outcomes	Duration of treatment
**ACEi/ARB trials**										
Leonetti 1991 [9]	Italy	36	66	72	Older people with hypertension	Captopril 25-50mg twice daily	Placebo	Bicycle endurance exercise time	None	2 months
Gerdts 2006 [10]	Norway	51	68	49	55-80 years with hypertension and LVH on ECG	Losartan 50-100mg once daily + HCTZ if required	Atenolol 50-100mg once daily + HCTZ if required	VO_2max	Maximum load (W)	1 year
Sumukadas 2007 [11]	Scotland	130	79	71	65 and over with impairment of ADLs	Perindopril 2-4mg once daily	Placebo	6MWD	TUAG<br>10-rep STS	20 weeks
Bunout 2009 [12]	Chile	120	75	76	70 and over with stage I hypertension	Enalapril 10-20mg once daily + HCTZ if required	Nifedipine slow-release 20mg once daily	12MWD	Handgrip strength<br>Quadriceps strength<br>SPPB<br>TUAG	9 months
Cesari 2010 [13]	USA	294	66	42	55 and over with elevated cardiovascular risk	Fosinopril 20-40mg once daily	Placebo	Rescaled SPPB	Handgrip strength	6 months
Sumukadas 2013 [14]	Scotland	170	76	42	65 and over with SPPB ≤ 10	Perindopril 2-4mg once daily + mixed modality exercise training	Placebo + mixed modality exercise training	6MWD	SPPB<br>Quadriceps strength<br>Handgrip strength	20 weeks
Sumukadas 2018 [15]	Scotland	80	78	75	65 and over with >1 self-reported fall in last 12 months	Perindopril 2-4mg once daily	Placebo	Postural sway	6MWD<br>Quadriceps strength	15 weeks
Heisterberg 2018 [16]	Denmark	71	72	0	Healthy, untrained males without hypertension or other disease	Losartan 50-100mg once daily + resistance training	Placebo + resistance training	Quadriceps mass	Isometric Quadriceps strength<br>Isokinetic quadriceps strength	16 weeks
**Leucine trials**										
Verhoeven 2009 [17]	Netherlands	30	71	0	Healthy older men	Leucine 2.5g three times a day	Placebo	Leg press strength	Fat free mass<br>Leg extension strength<br>Insulin resistance	12 weeks
Leenders 2011 [18]	Netherlands	60	71	0	Older men with type 2 diabetes mellitus	Leucine 2.5g three times a day	Placebo	Leg press strength	Fat free mass<br>Leg extension strength	6 months

									Insulin resistance	
Ispoglou 2016 [19]	England	25	72	56	Healthy non-smokers aged 65-75	Essential amino acid mix with 0.08g/kg/day leucine	Essential amino acid mix with 0.04 g/kg/day leucine	Lean body mass	6MWD<br>Chair stand test<br>Arm curl test<br>Handgrip strength	12 weeks
Martinez-Arnau 2020 [20]	Spain	50	79	67	Nursing home residents aged 65 and over	Leucine 3g twice a day	Placebo	Handgrip strength	Walking time<br>Whole body muscle mass<br>Calf and arm circumference<br>Peak flow	13 weeks

SPPB: Short Physical Performance Battery. TUAG: Timed Up And Go test. 6MWD: Six minute walk distances. 12MWD: Twelve minute walk distance. VO_2max: Maximal oxygen uptake. STS: Sit to stand test.

Supplementary Figure 1: Flowchart for perindopril uptitration

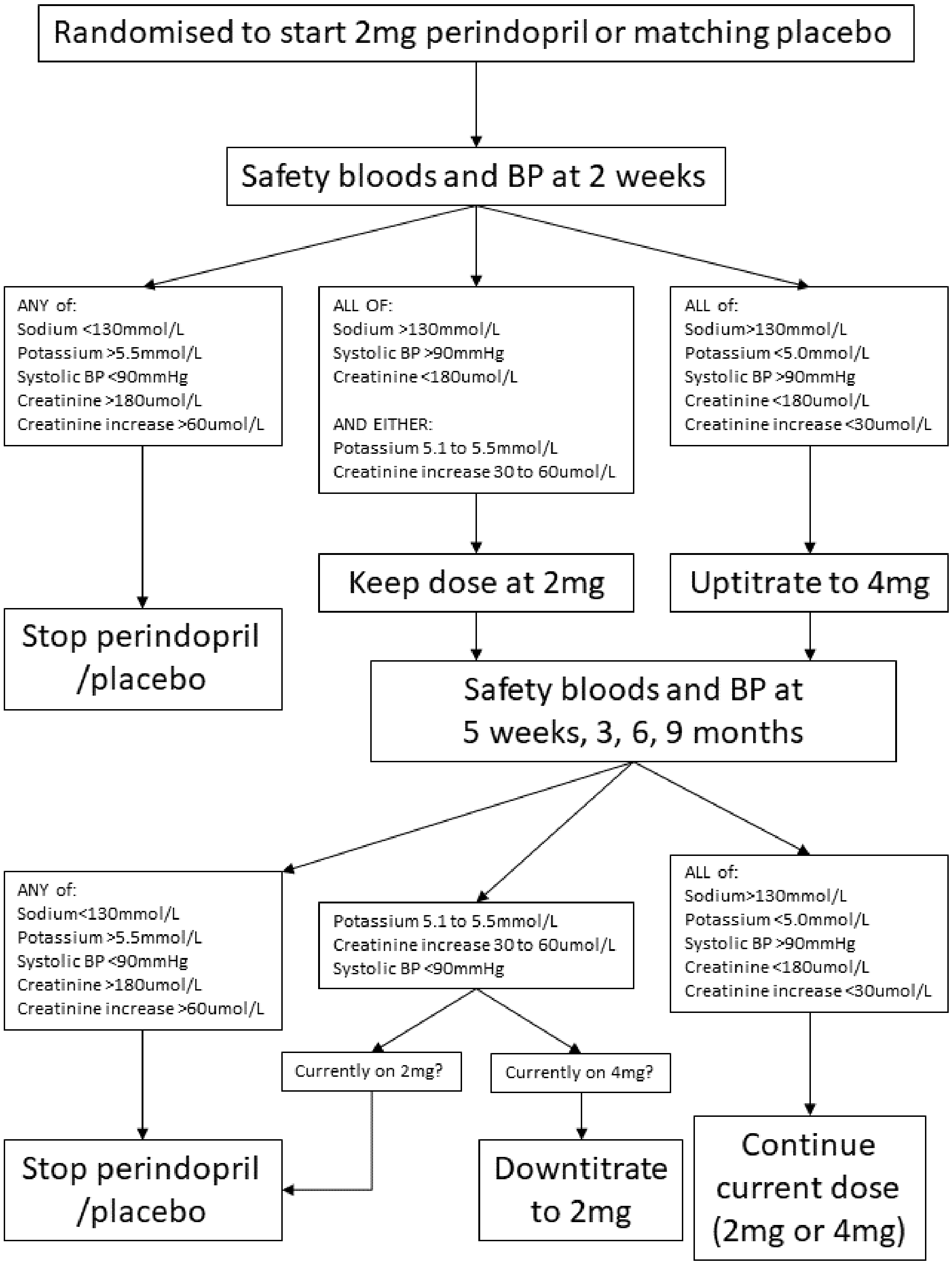

Supplementary Figure 2: Change in lying blood pressure and postural blood pressure drop for perindopril vs placebo analysis

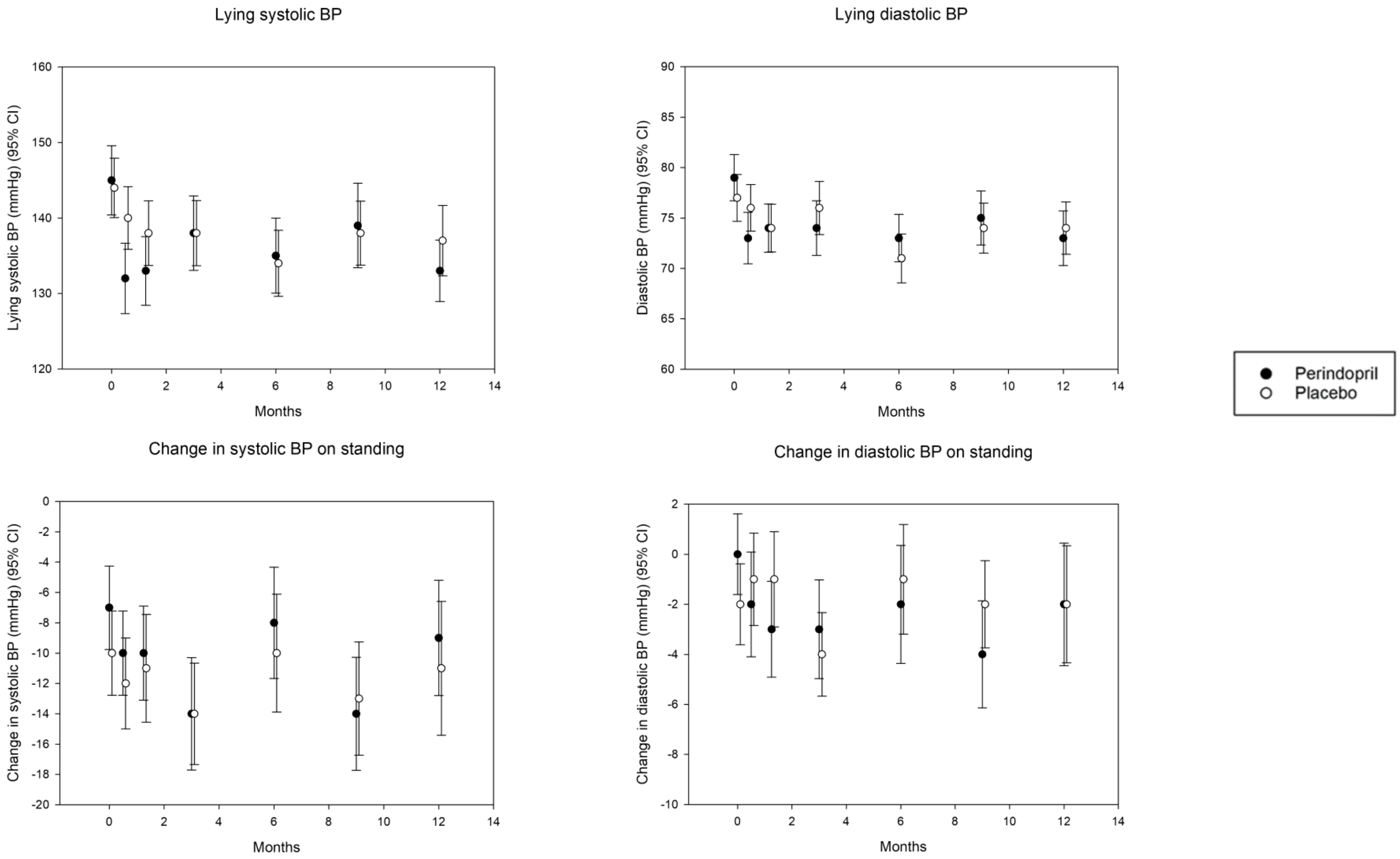

Supplementary Figure 3. Meta-analysis Forest plots

a. Effect of ACEi/ARB on Short Physical Performance Battery

	ACEi/ARB			Control				Mean Difference
**Study or Subgroup**	**Mean**	**SD**	**Total**	**Mean**	**SD**	**Total**	**Weight**	**IV, Random, 95% CI**
Bunout 2009	0.1	2	58	0.5	1.8	49	21.0%	-0.40 [-1.12, 0.32]
Cesari 2010	2.11	3.075	225	2.1	3.075	225	33.8%	0.01 [-0.56, 0.58]
LACE 2021	0.04	2.3069	73	0	2.291	72	19.5%	0.04 [-0.71, 0.79]
Sumukadas 2013	1	2.2	83	1.1	2	77	25.7%	-0.10 [-0.75, 0.55]
**Total (95% CI)**			**439**			**423**	**100.0%**	**-0.10 [-0.43, 0.23]**

Heterogeneity: Tau² = 0.00; Chi² = 0.94, df = 3 (P = 0.81); I² = 0%
Test for overall effect: Z = 0.59 (P = 0.56)

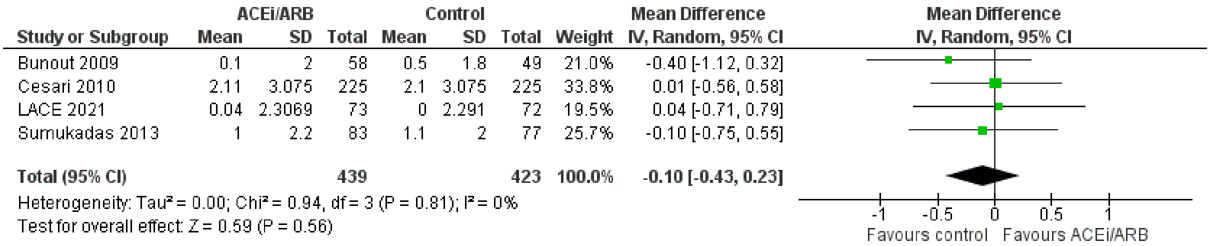

b. Effect of ACEi/ARB on six-minute walk distance (in metres)

	ACEi/ARB			Control				Mean Difference	
**Study or Subgroup**	**Mean**	**SD**	**Total**	**Mean**	**SD**	**Total**	**Weight**	**IV, Random, 95% CI**	**Year**
Sumukadas 2007	24.6	51	45	-6.8	49	50	29.8%	31.40 [11.24, 51.56]	2007
Sumukadas 2013	20.6	74.9	83	36.4	59.2	77	29.5%	-15.80 [-36.64, 5.04]	2013
Sumukadas 2018	2	99	38	21	112	38	17.3%	-19.00 [-66.53, 28.53]	2018
LACE 2021	-7	102	73	0	102	72	23.4%	-7.00 [-40.21, 26.21]	2021
**Total (95% CI)**			**239**			**237**	**100.0%**	**-0.24 [-27.95, 27.47]**	

Heterogeneity: Tau² = 565.55; Chi² = 11.88, df = 3 (P = 0.008); I² = 75%
Test for overall effect: Z = 0.02 (P = 0.99)

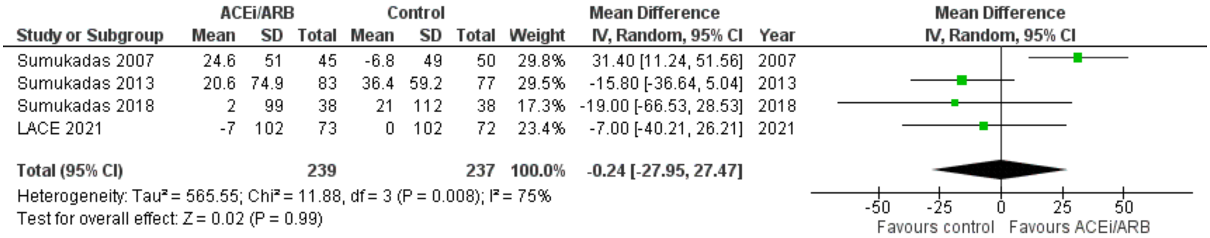

c. Effect of ACEi/ARB on handgrip strength (in kg)

	ACEi/ARB			Control				Mean Difference	
**Study or Subgroup**	**Mean**	**SD**	**Total**	**Mean**	**SD**	**Total**	**Weight**	**IV, Random, 95% CI**	**Year**
Bunout 2009	-0.4	7.2	58	-0.3	6.4	49	11.3%	-0.10 [-2.68, 2.48]	2009
Cesari 2010	36.9	9.2	225	37	9.2	225	25.9%	-0.10 [-1.80, 1.60]	2010
Sumukadas 2013	0.6	3.3	83	1.1	4.6	77	48.0%	-0.50 [-1.75, 0.75]	2013
LACE 2021	0.8	6.9	73	0	6.9	72	14.8%	0.80 [-1.45, 3.05]	2021
**Total (95% CI)**			**439**			**423**	**100.0%**	**-0.16 [-1.02, 0.71]**	

Heterogeneity: Tau² = 0.00; Chi² = 0.99, df = 3 (P = 0.80); I² = 0%
Test for overall effect: Z = 0.36 (P = 0.72)

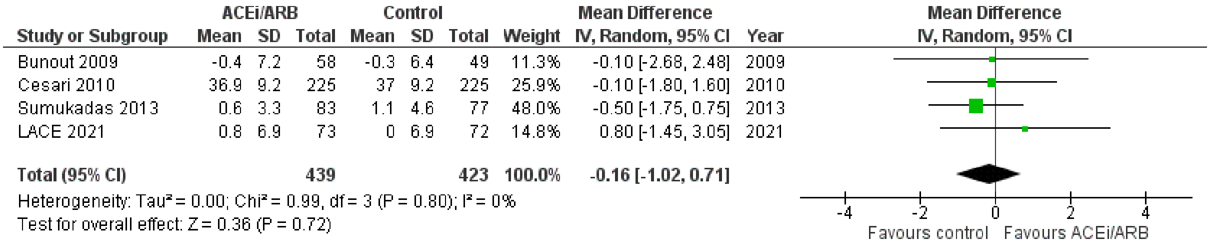

d. Effect of ACEi/ARB on quadriceps strength (in kg)

	ACEi/ARB			Control				Mean Difference	
**Study or Subgroup**	**Mean**	**SD**	**Total**	**Mean**	**SD**	**Total**	**Weight**	**IV, Random, 95% CI**	**Year**
Bunout 2009	0	8.3	58	0.4	7.8	49	13.8%	-0.40 [-3.45, 2.65]	2009
Sumukadas 2013	0.1	12.5	83	2	14.8	77	7.1%	-1.90 [-6.16, 2.36]	2013
Heisterberg 2018	36.9	25.9	20	31.4	23.3	18	0.5%	5.50 [-10.14, 21.14]	2018
Sumukadas 2018	-1.9	3.6	36	-0.6	3.5	35	47.1%	-1.30 [-2.95, 0.35]	2018
LACE 2021	-0.1	6.2	73	0	6.2	72	31.5%	-0.10 [-2.12, 1.92]	2021
**Total (95% CI)**			**270**			**251**	**100.0%**	**-0.80 [-1.94, 0.33]**	

Heterogeneity: Tau² = 0.00; Chi² = 1.76, df = 4 (P = 0.78); I² = 0%
Test for overall effect: Z = 1.39 (P = 0.16)

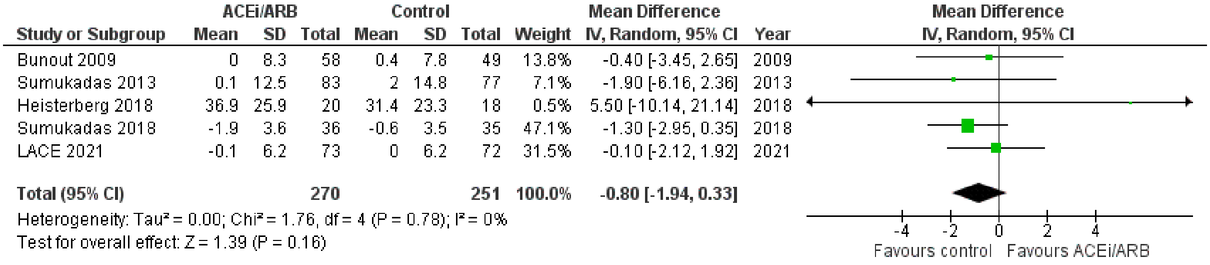

e. Effect of Leucine on six-minute walk distance (in metres)

Study or Subgroup	Leucine Mean	Leucine SD	Leucine Total	Control Mean	Control SD	Control Total	Weight	Mean Difference IV, Random, 95% CI
Ispoglou 2016	28	77	8	37	92	8	13.8%	-9.00 [-92.13, 74.13]
LACE 2021	6	102	72	0	102	73	86.2%	6.00 [-27.21, 39.21]
**Total (95% CI)**			**80**			**81**	**100.0%**	**3.94 [-26.90, 34.77]**

Heterogeneity: Tau² = 0.00; Chi² = 0.11, df = 1 (P = 0.74); I² = 0%
Test for overall effect: Z = 0.25 (P = 0.80)

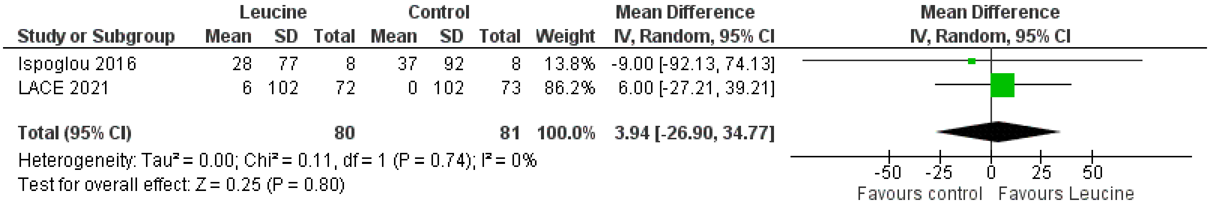

f. Effect of Leucine on walk speed (in m/s)

Study or Subgroup	Leucine Mean	Leucine SD	Leucine Total	Control Mean	Control SD	Control Total	Weight	Mean Difference IV, Random, 95% CI	Year
Martinez-Arnau 2020	-0.01	0.43	23	-0.1	0.53	19	25.3%	0.09 [-0.21, 0.39]	2020
LACE 2021	-0.03	0.53	72	0	0.53	73	74.7%	-0.03 [-0.20, 0.14]	2021
**Total (95% CI)**			**95**			**92**	**100.0%**	**0.00 [-0.15, 0.15]**	

Heterogeneity: Tau² = 0.00; Chi² = 0.47, df = 1 (P = 0.49); I² = 0%
Test for overall effect: Z = 0.01 (P = 1.00)

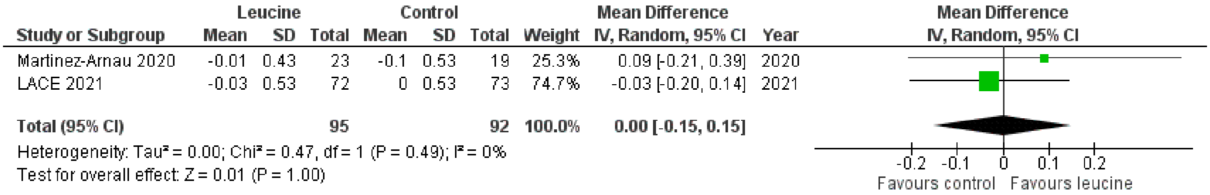

g. Effect of Leucine on handgrip strength (in kg)

Study or Subgroup	Leucine Mean	Leucine SD	Leucine Total	Control Mean	Control SD	Control Total	Weight	Mean Difference IV, Random, 95% CI	Year
Ispoglou 2016	0.8	9.8	8	1.5	7.5	8	5.2%	-0.70 [-9.25, 7.85]	2016
Martinez-Arnau 2020	0.1	9.7	23	-0.9	8.7	19	12.3%	1.00 [-4.57, 6.57]	2020
LACE 2021	-0.6	6.6	72	0	6.6	73	82.5%	-0.60 [-2.75, 1.55]	2021
**Total (95% CI)**			**103**			**100**	**100.0%**	**-0.41 [-2.36, 1.54]**	

Heterogeneity: Tau² = 0.00; Chi² = 0.28, df = 2 (P = 0.87); I² = 0%
Test for overall effect: Z = 0.41 (P = 0.68)

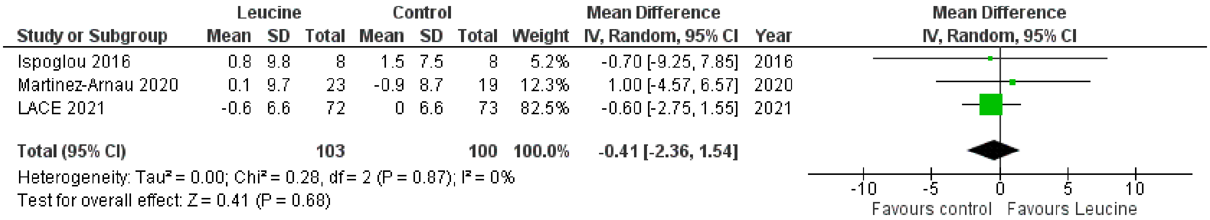

h. Effect of Leucine on quadriceps strength (in kg)

Study or Subgroup	Leucine Mean	Leucine SD	Leucine Total	Control Mean	Control SD	Control Total	Weight	Mean Difference IV, Random, 95% CI	Year
Leenders 2011	4	14	29	6	19	28	5.1%	-2.00 [-10.69, 6.69]	2011
LACE 2021	-0.6	6.2	72	0	6.2	73	94.9%	-0.60 [-2.62, 1.42]	2021
**Total (95% CI)**			**101**			**101**	**100.0%**	**-0.67 [-2.64, 1.29]**	

Heterogeneity: Tau² = 0.00; Chi² = 0.09, df = 1 (P = 0.76); I² = 0%
Test for overall effect: Z = 0.67 (P = 0.50)

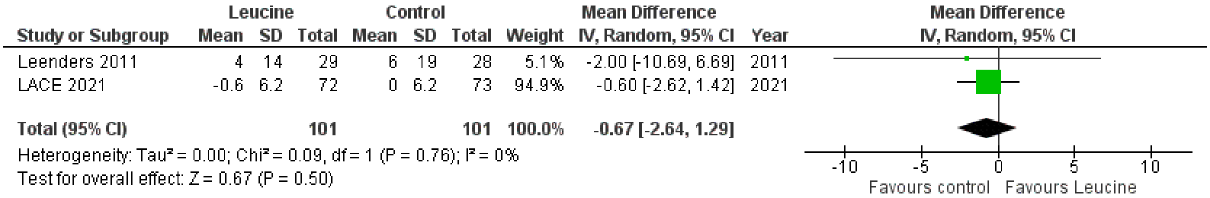

i. Effect of Leucine on lean body mass (z scores)

Study or Subgroup	Leucine Mean	Leucine SD	Leucine Total	Control Mean	Control SD	Control Total	Weight	Std. Mean Difference IV, Random, 95% CI	Year
Verhoeven 2009	0.4	4.9	15	0.4	3.8	14	10.0%	0.00 [-0.73, 0.73]	2009
Leenders 2011	0.1	5.6	29	0	6.9	28	19.8%	0.02 [-0.50, 0.54]	2011
Ispoglou 2016	0.5	9	8	0	7.2	8	5.5%	0.06 [-0.92, 1.04]	2016
Martinez-Arnau 2020	-0.2	1.8	23	0.3	3.4	19	14.4%	-0.19 [-0.79, 0.42]	2020
LACE 2021	0.2	1.5	72	0.25	1.5	73	50.3%	-0.03 [-0.36, 0.29]	2021
**Total (95% CI)**			**147**			**142**	**100.0%**	**-0.04 [-0.27, 0.19]**	

Heterogeneity: Tau² = 0.00; Chi² = 0.31, df = 4 (P = 0.99); I² = 0%
Test for overall effect: Z = 0.31 (P = 0.75)

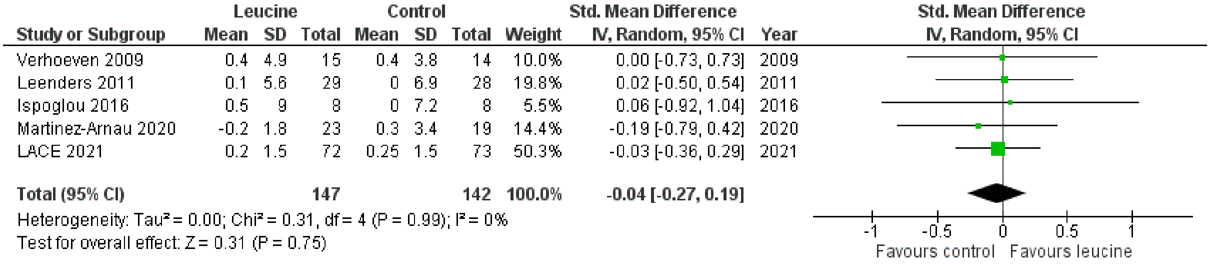

References for Supplementary Material

[1] Roberts HC, Denison HJ, Martin HJ, Patel HP, Syddall H, Cooper C et al. A review of the measurement of grip strength in clinical and epidemiological studies: towards a standardised approach. Age Ageing 2011;40:423-9.

[2] Mentiplay BF, Perraton LG, Bower KJ, Adair B, Pua YH, Williams GP et al. Assessment of Lower Limb Muscle Strength and Power Using Hand-Held and Fixed Dynamometry: A Reliability and Validity Study. PLoS One 2015;10:e0140822.

[3] Guyatt GH, Sullivan MJ, Thompson PJ, Fallen EL, Pugsley SO, Taylor DW et al. The 6-minute walk: a new measure of exercise capacity in patients with chronic heart failure. Can Med Assoc J 1985;132:919-23.

[4] Wennie Huang WN, Perera S, Vanswearingen J, Studenski S. Performance measures predict onset of activity of daily living difficulty in community-dwelling older adults. J Am Geriatr Soc 2010;58:844-52.

[5] Bohannon RW. Reference values for the timed up and go test: a descriptive meta-analysis. J Geriatr Phys Ther 2006;29:64-8.

[6] Nouri FM, Lincoln NB. An extended activities of daily living scale for stroke patients. Clin Rehabilitation 1987;1:301-5.

[7] EuroQol Group EuroQol--a new facility for the measurement of health-related quality of life. Health Policy 1990;16:199–208.

[8] Matthews DR, Hosker JP, Rudenski AS, Naylor BA, Treacher DF, Turner RC. Homeostasis model assessment: insulin resistance and beta-cell function from fasting plasma glucose and insulin concentrations in man. Diabetologia 1985;28:412–419

[9] Leonetti G, Mazzola C, Pasotti C, Angioni L, Vaccarella A, Capra A et al. Treatment of hypertension in the elderly: Effects on blood pressure, heart rate, and physical fitness. Am J Med 1991;90:12S-13S.

[10] Gerdts E, Björnstad H, Devereux RB, Lund-Jhansen P, Davidsen ES, Omvik P. Exercise performance during losartan- or atenolol-based treatment in hypertensive patients with electrocardiographic left ventricular hypertrophy (a LIFE substudy). Blood Press 2006;15:220-6.

[11] Sumukadas D, Witham MD, Struthers AD, McMurdo MET. Effect of perindopril on physical function in elderly people with functional impairment: a randomised controlled trial. CMAJ 2007; 177: 867-74.

[12] Bunout D, Barrera G, de la Maza MP, Leiva L, Backhouse C, Hirsch S. Effects of enalapril or nifedipine on muscle strength or functional capacity in elderly subjects. A double blind trial. J Renin Angiotensin Aldosterone Syst 2009;10:77-84.

[13] Cesari M, Pedone C, Incalzi RA, Pahor M. ACE-inhibition and physical function: Results from the Trial of Angiotensin-Converting Enzyme Inhibition and Novel Cardiovascular Risk Factors (TRAIN) study. J Am Med Dir Assoc 2010;11:26-32.

[14] Sumukadas D, Band M, Miller S, Cvoro V, Witham MD, Struthers AD, McConnachie A, Lloyd SM, McMurdo ME. Do ACE inhibitors improve the response to exercise training in functionally impaired older adults?: a randomised controlled trial. J Gerontol A Med Sci 2013;69:736-43.

[15] Sumukadas D, Price R, McMurdo MET, Rauchhaus P, Struthers A, McSwiggan S et al. The effect of perindopril on postural instability in older people with a history of falls—a randomised controlled trial. Age Ageing 2018;47:75-81.

[16] Heisterberg MF, Andersen JL, Schjerling P, Lund A, Dalskov S, Jønsson AO et al. Losartan has no additive effect on the response to heavy-resistance exercise in human elderly skeletal muscle. J Appl Physiol (1985). 2018;125:1536-54.

[17] Verhoeven S, Vanschoonbeek K, Verdijk LB, Koopman R, Wodzig WKWH, Dendale P et al. Long-term leucine supplementation does not increase muscle mass or strength in healthy elderly men. Am J Clin Nutr 2009;89:1468-75.

[18] Leenders M, Verdijk LB, van der Hoeven L, van Kranenburg J, Hartgens F, Wodzig WK et al. Prolonged leucine supplementation does not augment muscle mass or affect glycemic control in elderly type 2 diabetic men. J Nutr 2011;141:1070-6.

[19] Ispoglou T, White H, Preston T, McElhone S, McKenna J, Hind K. Double-blind, placebo-controlled pilot trial of L-Leucine-enriched amino-acid mixtures on body composition and physical performance in men and women aged 65-75 years. Eur J Clin Nutr 2016;70:182-8.

[20] Martínez-Arnau FM, Fonfría-Vivas R, Buigues C, Castillo Y, Molina P, Hoogland AJ et al. Effects of Leucine Administration in Sarcopenia: A Randomized and Placebo-controlled Clinical Trial. Nutrients 2020;12:932.
